# Recent advances in multimodality imaging‐guided therapy in pericarditis

**DOI:** 10.1111/eci.70067

**Published:** 2025-05-23

**Authors:** Joseph El Roumi, Aldo L. Schenone, Paul Cremer, Tom Kai Ming Wang, Allan Klein

**Affiliations:** ^1^ Center for the Diagnosis and Treatment of Pericardial Diseases, Section of Cardiovascular Imaging, Robert and Suzanne Tomsich Department of Cardiovascular Medicine Sydell and Arnold Miller Family Heart, Vascular, and Thoracic Institute, Cleveland Clinic Cleveland Ohio USA; ^2^ Division of Cardiology Montefiore Einstein Center for Heart and Vascular Care. Albert Einstein College of Medicine Bronx New York USA; ^3^ Department of Medicine, Division of Cardiology Bluhm Cardiovascular Institute, Northwestern University Feinberg School of Medicine Chicago Illinois USA

**Keywords:** cardiac CT, cardiac magnetic resonance, echocardiography, multimodality imaging, pericardial constriction, pericarditis

## Abstract

**Background:**

In the era of precision medicine, cardiac multimodality imaging plays a vital role in diagnosing, managing, and monitoring pericarditis. This condition, often marked by inflammation and recurrent episodes, requires precise imaging techniques to guide diagnosis and therapeutic decisions.

**Methods:**

We carefully reviewed the medical literature for high‐quality data regarding the use of multimodality imaging in pericarditis and the precious value of the novel concept of imaging‐guided therapy.

**Results:**

While echocardiography remains the cornerstone for detecting pericardial effusion and evaluating hemodynamics, its limited ability to characterize inflammation has driven the use of advanced modalities such as cardiac magnetic resonance imaging (CMR), cardiac computed tomography (CT), and positron emission tomography (PET). CMR offers superior visualization of pericardial inflammation through late gadolinium enhancement, aiding in identifying patients who may benefit from targeted anti‐inflammatory therapies. CT imaging, with its high spatial resolution, aids in detecting pericardial calcifications and thickening, particularly in constrictive pericarditis. PET, often combined with CT, is a valuable tool for quantifying metabolic activity, allowing the detection of active inflammation, particularly in complex or refractory cases. Multiple imaging targets have been identified as essential biomarkers to confirm the inflammatory phenotype, assess treatment response, and monitor for complications.

**Conclusion:**

Considering the inherent limitations of each imaging modality, the integration of imaging findings with clinical and biomarker data may aid clinicians in tailoring therapy according to different clinical scenarios and better stratification of patients who may benefit from IL‐1 blockade. This review explores the valuable role of cardiac multimodality imaging‐guided therapy in managing pericarditis.

## Graphical abstract

Multiple imaging targets have been identified as essential biomarkers to confirm pericardial thickening, edema, neovascularization, inflammation, fibrosis/calcification, cardiac tamponade physiology, and constriction. This novel approach is called imaging‐guided therapy, which guides anti‐inflammatory therapy, assesses treatment response, assists in the longitudinal follow‐up of patients, and is also useful for monitoring for complications. By integrating imaging findings with clinical and biomarker data, clinicians can also better stratify patients who may benefit from IL‐1 blockade, optimizing outcomes while minimizing overtreatment.

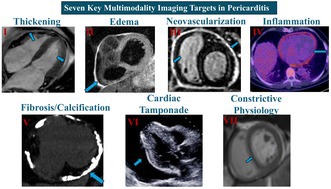

## 1 INTRODUCTION

Pericardial diseases are the second most frequent cause of acute care visits for chest pain, with pericarditis accounting for approximately 5% of all emergency department presentations for non‐ischemic chest pain in developed countries.^1^ The clinical diagnosis and management of acute pericarditis remain challenged by the suboptimal sensitivity and specificity of conventional diagnostic approaches, limitations in accurately distinguishing active inflammation from chronic fibrosis, and the lack of standardized protocols for monitoring therapeutic response.

These gaps in diagnosis and management underscore the increasing importance of cardiac multimodality imaging (cMMI) as a valuable tool for managing patients with known or suspected pericarditis.^2^, ^3^, ^4^ While transthoracic echocardiography (TTE) remains the first‐line imaging modality for the initial assessment, increasing evidence supports the essential role of cardiac magnetic resonance imaging (CMR) in cases of diagnostic uncertainty or complicated pericarditis. Advanced imaging techniques, such as CMR, hybrid 18F‐fluorodeoxyglucose positron emission tomography (18F‐FDG PET) imaging, or cardiac computed tomography (CT), offer superior capabilities in evaluating pericardial thickness, identifying pericardial inflammation, and ruling out constrictive physiology.^3^, ^5^ This review provides a comprehensive overview of pericarditis, encompassing its underlying pathophysiology and diverse clinical presentations. It also provides a practical framework for applying cMMI in diagnosing and managing pericarditis across its entire spectrum of presentation.

## 2 EPIDEMIOLOGY AND CLINICAL PRESENTATION OF PERICARDITIS

Acute pericarditis (AP) has an estimated annual incidence of 27.7 cases per 100,000, with a higher prevalence in males aged 16–65 years.^1^, ^6^ Table 1 summarizes the diagnostic criteria of pericarditis, defines major subtypes, and describes the adverse sequelae of complicated AP. In developed countries, 80%–90% of AP cases are idiopathic or post‐viral, with 70%–85% of patients achieving clinical remission (symptoms resolution and no recurrence) with non‐steroidal anti‐inflammatory drugs (NSAIDs) and colchicine.^3^, ^7^ Tuberculosis (TB) is a significant aetiology in developing regions, depending on endemicity.^6^, ^8^ Post‐cardiac injury syndrome following cardiac surgery and procedures, including electrophysiological ablations and percutaneous coronary interventions, is increasingly recognized as a cause of AP.

**TABLE 1:** Diagnostic Criteria of Pericarditis and Its Associated Subtypes.

Pericarditis subtypes	Diagnostic criteria
Acute Pericarditis (AP)	**At least two of the following must be present:**
	Chest pain: Typically sharp, pleuritic, and relieved by sitting forward.
	Pericardial friction rub: High‐pitched, scratchy sound best heard with the diaphragm of the stethoscope at the left lower sternal border.
	ECG changes: Widespread ST‐segment elevation and/or PR‐segment depression not attributable to ischemia.
	Pericardial effusion: New or worsening effusion detected by echocardiography or imaging.
	Supportive findings (may strengthen the diagnosis but are not required):
	Elevated inflammatory markers (CRP, WSR, leukocytosis).
	Evidence of pericardial inflammation on imaging (e.g., MRI showing late gadolinium enhancement).
Recurrent Pericarditis	Recurrence of pericarditis after a symptom‐free interval of >4–6 weeks following an initial episode of AP.
	Diagnosis requires:
	A documented initial episode of AP.
	At least one of the following signs of recurrence:
	Recurrent chest pain typical of pericarditis.
	Pericardial friction rub.
	Pericardial effusion (new or worsening).
	Recurrent ECG changes typical of pericarditis.
	Elevated inflammatory markers (ESR, CRP).
Incessant Pericarditis	Persistent pericarditis lasting >4–6 weeks without resolution.
	Symptoms and objective signs of pericardial inflammation continuously present.
	Incessant pericarditis lacks a symptom‐free interval.
Effusive Constrictive Pericarditis	Presence of concurrent pericardial effusion and features of constrictive pericarditis (CP).
	Hemodynamic findings of CP (e.g., ventricular interdependence, respiratory variation in mitral/tricuspid inflow velocities).
	Failure of pericardial effusion drainage to resolve CP.
	Supported by imaging (echocardiography, CT, or MRI) showing thickened pericardium and persistent effusion.
Transient Constrictive Pericarditis	Temporary constrictive physiology with following pericardial inflammation.
	Typically resolves within 3–6 months with anti‐inflammatory therapy.
	Diagnostic features of constrictive pericarditis but with documented resolution over time.
	Imaging (e.g., echocardiography, CT, or MRI) showing initial pericardial thickening with subsequent normalization.

*Note*: It underscores the major criteria used in the diagnosis of acute pericarditis (AP) and associated subtypes and complicated disease states. The red asterisks in the ECG and accompanying TTE parasternal short‐axis view highlight diffuse ST‐segment elevations and a small pericardial effusion, respectively. The red arrow in the middle CMR figure demarcates increased pericardial signal intensity on T2‐STIR weighted imaging, consistent with pericardial edema, as seen in cases of recurrent pericarditis. The white asterisk in the bottom TTE figure shows a pericardial effusion, while the cardiac catheterization tracing shows ventricular interdependence on simultaneous right and left pressure waveforms, used in the hemodynamic assessment and diagnosis of pericardial constriction.

Pericarditis encompasses a spectrum of conditions, from a non‐inflammatory phenotype (normal or near‐normal C‐reactive protein) to a distinctly inflammatory one. The inflammatory phenotype is characterized by high fever, elevated CRP, and neutrophilia and is often accompanied by pericardial effusion (PEff) and pleural involvement.^9^, ^10^ Recurrent and incessant pericarditis may also present with non‐inflammatory (less common) or inflammatory (more common) phenotypes.^11^ Myocardial involvement as either myo‐pericarditis (predominantly pericarditis) or peri‐myocarditis (predominantly myocarditis) is not infrequent, occurring in up to 30% of patients.^12^, ^13^ Complicated pericarditis significantly impairs quality of life, functional capacity, and mental health,^1^, ^3^, ^14^, ^15^ with episode frequency and duration contributing substantially to overall morbidity.^3^ PEff, cardiac tamponade (CTP), and constrictive pericarditis (CP) are potential complications of acute pericarditis (AP). PEff is present in up to 60% of idiopathic AP patients at diagnosis, with a 5%–15% risk of cardiac tamponade (CTP). CTP risk is elevated in neoplastic, infectious (fungal, bacterial, Human Immunodeficiency Virus‐associated), and tuberculous AP.^2^, ^16^ Though rare (<.5% in idiopathic/viral AP), CP is more frequent in bacterial/tuberculous pericarditis and occurs in ~20% of patients with incessant pericarditis.^1^, ^17^, ^18^

## 3 PERICARDIAL RESPONSE TO INJURY AND PERICARDIAL INFLAMMATION

Pericardial inflammation, or pericarditis, is triggered by injury to the pericardial mesothelial cells. Common etiologies of injury include viral infections, cardiac surgery, ablation procedures, autoinflammatory processes, and autoimmune syndromes.^1^, ^2^ Tissue injury or infectious agents release pathogen‐associated molecular patterns (PAMPs) or damage‐associated molecular patterns (DAMPs), which are endogenous danger signals. These activate Toll‐like receptor activation, inducing nuclear factor kappa B (NF‐κB) synthesis and assembly of the NLRP3 inflammasome complex.^14^ This results in the release of interleukins (IL‐1α and IL‐1β) and other proinflammatory cytokines, which amplify and perpetuate the proinflammatory response.^14^, ^19^

In response to a pathologic insult and inflammatory response, vascular permeability is increased along with neovascularization of the pericardium, leading to fluid, fibrin, and inflammatory cell exudation with mesothelial cell desquamation.^1^, ^3^, ^20^, ^21^ The remission phase involves fibroblast proliferation and the formation of granulation tissue. Repetitive inflammatory insults to the pericardium may induce pericardial thickening, fibrosis, and calcification, which may herald the development of constrictive pericarditis (CP).^2^, ^22^

## 4 IMAGING TARGETS IN PERICARDITIS

**FIGURE 1:**
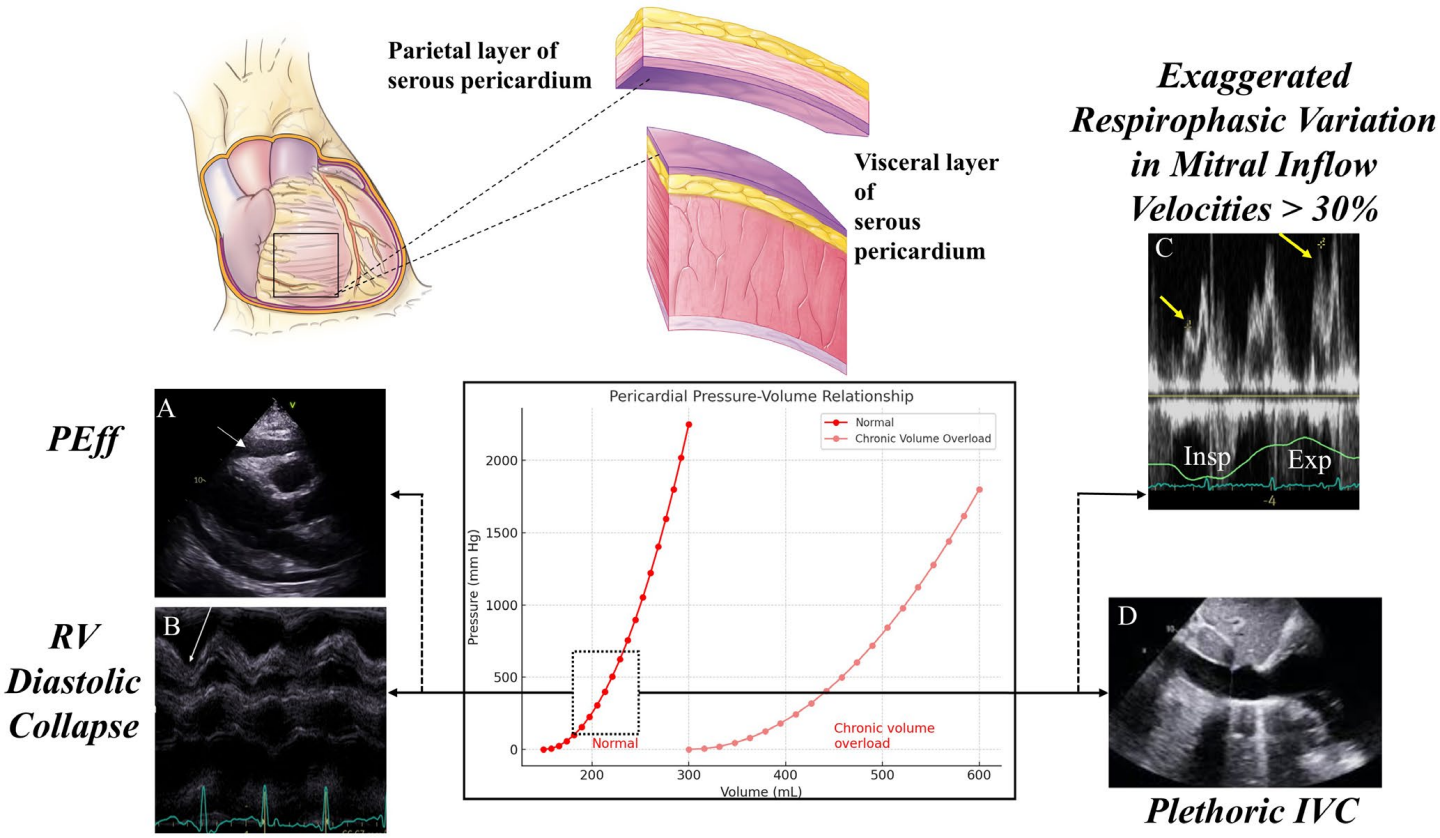
Anatomical representation of the pericardium, pressure‐volume relationship curve, and echocardiographic findings characteristic of cardiac tamponade. It highlights pericardial histology and normal pericardial pressure‐volume curve (dark red curve) and in a chronic volume overload state (light red curve). The dotted rectangle in the graph underscores the critical phase in a healthy individual, where small increases in pericardial volume translate into accentuated elevation in intrapericardial pressures. This process can ultimately lead to pericardial tamponade, with major echocardiographic findings highlighted in the accompanying echocardiographic imaging (A–D). (A) Parasternal long‐axis (PLAX) view with arrow highlighting circumferential pericardial effusion (PEff). (B) M‐mode across PLAX view (not shown) revealing diastolic right ventricular collapse, a hallmark sign of cardiac tamponade (CTP). (C) Pulsed‐wave (PW) Doppler of mitral inflow showing >30% variation in mitral inflow velocities during respiration. Noted is an increase in mitral inflow during expiration, compared to inflow during the inspiratory phase. (D) Subcostal image depicting dilated (>2.1 cm) and plethoric inferior vena cava (IVC).

The pericardium is a thin (<1 mm), relatively avascular, and double‐layered sac surrounding the heart. A small volume (<50 mL) of serous fluid lubricates the pericardial space between these layers. The parietal pericardium is the outer, thicker layer, composed of a relatively inelastic fibrous layer (fibrosa) lined by a serous membrane of mesothelial cells. The visceral pericardium is the inner serous membrane, directly adherent to the epicardium. The pericardium restricts chamber dilation and influences the heart's passive pressure‐volume relationship.^2^, ^20^, ^23^, ^24^ The distensibility of the normal pericardium is primarily governed by collagen fibres, which are wavy at low strain but straighten under tension. Thus, normal parietal pericardial tissue exhibits a stress–strain curve with a flat response at low stress, becoming markedly steeper at higher stress^2^, ^25^ (Figure 1).

**FIGURE 2:**
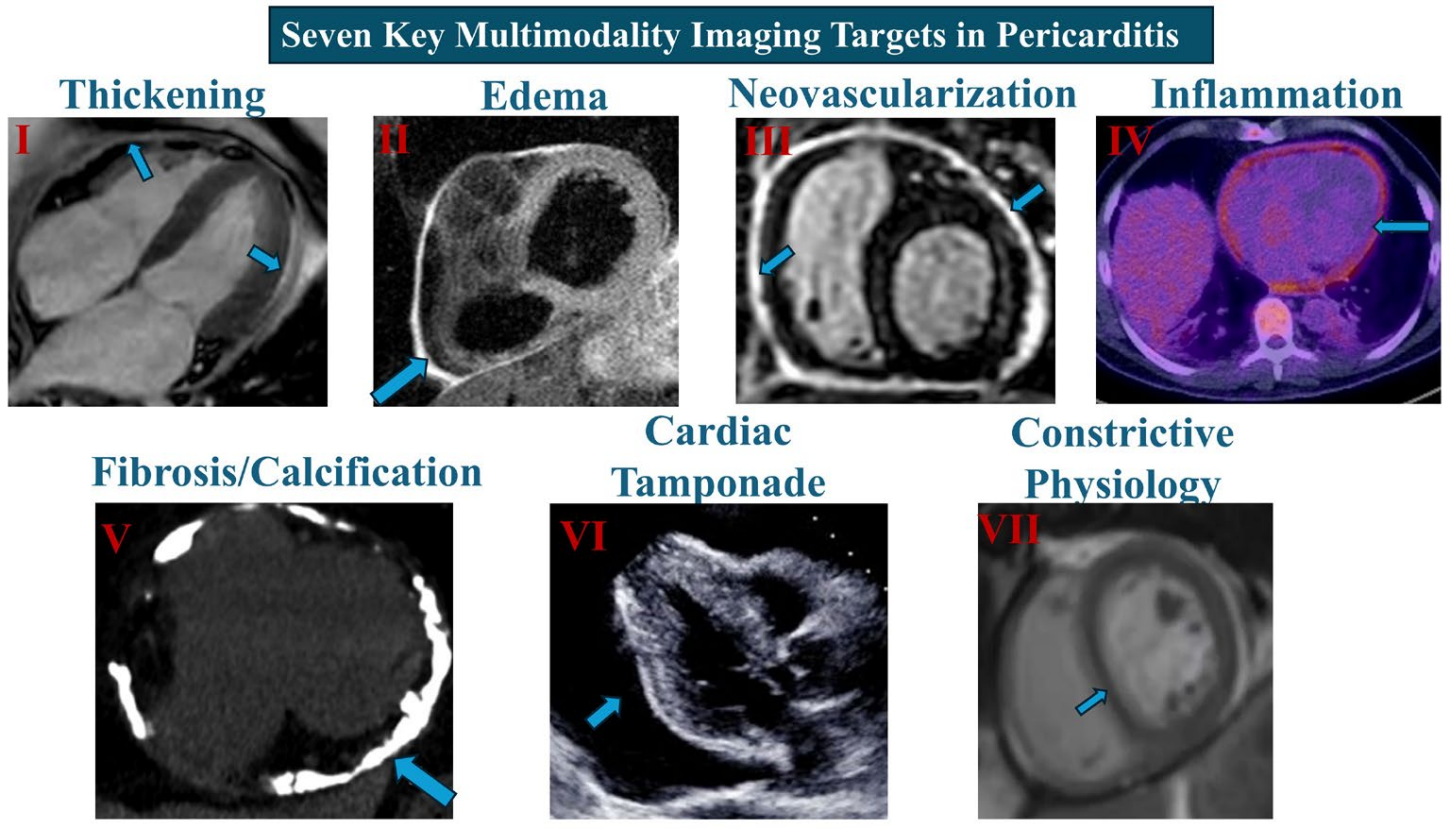
It depicts the 7 key imaging targets (roman numerals) that should be evaluated in patients presenting with pericarditis, with examples from multimodality imaging: I. Thickening: CMR cine‐4 chamber showing significantly thickened pericardium. II. Edema: Increased pericardial signal intensity on T2‐STIR weighted CMR imaging. III. Neovascularization: Circumferential LGE which is a surrogate for pericardial neovascularization and inflammation injury. IV. Inflammation: FDG‐PET CT showing diffuse circumferential pericardial uptake representing edematous and inflamed pericardium. TTE apical 4‐chamber view showing circumferential pericardial effusion. V. Fibrosis and Calcification: Cardiac CT showing extensive and almost circumferential pericardial calcifications. VI. Cardiac Tamponade Physiology (CTP): TTE parasternal long‐axis view showing large circumferential pericardial effusion. This patient had cardiac tamponade with evidence of right‐sided chamber diastolic collapse (not highlighted in this image). VII. Constrictive Physiology (CP): Real‐time free‐breathing sequence on CMR showing septal flattening as a sign of respirophasic septal shift indicating constrictive physiology.

Cardiac imaging plays a crucial role in the diagnosis, risk stratification, and management of patients with pericardial disease. A comprehensive understanding of the specific imaging targets in pericarditis is essential to characterising disease phenotype, determining severity and complications, and assessing response to therapy. Figure 2 highlights the seven key imaging targets critical to the assessment of pericarditis, supplemented by representative examples obtained through cMMI. By integrating key imaging findings, clinicians can diagnose more precisely, differentiate pericarditis subtypes, and optimise treatment strategies. Interpretation of CMR in pericarditis, particularly for markers such as LGE and T2‐weighted edema, is subject to notable inter‐reader variability. This variability can impact diagnostic accuracy and clinical decision‐making, especially in borderline or subtle cases. The absence of standardised criteria and the reliance on qualitative assessment further underscore the need for expert interpretation, ideally within a structured or core‐lab setting, to ensure reproducibility and consistency across centres.^26^

### 4.1 Pericardial Thickening

Normal pericardial thickness ranges near the spatial resolution limits of current cardiac imaging modalities and is either imperceptible or may appear as a paper‐thin linear structure. Thus, clear visualization of the normal pericardium on imaging presents a challenge, as anatomical studies indicate that the normal parietal pericardium is typically less than 1 mm in thickness. Pericardial thickening signals pericardial disease and may be appreciated with ongoing pericardial inflammation as in AP.^27^ It can also be visualized in the setting of fibrosis or calcification as sequelae of prior pericardial injury. Although pericardial thickening equates to pericardial disease, the presence of thickening is not a prerequisite for the diagnosis of pericarditis, as many cases of pericarditis, even up to 20% of patients with constrictive pericarditis, exhibit a normal pericardial thickness.^28^ Repeated episodes of pericarditis and prolonged inflammatory activity may lead to pericardial thickening, which can progress to a calcific or “burnt‐out” stage.^2^

While often used as a first‐line imaging modality, TTE has limitations in accurately evaluating pericardial thickness. Although TTE can identify severe pericardial thickening, it frequently fails to detect milder cases due to limited sensitivity and technical factors, such as transducer positioning and artefacts.^29^ Conversely, CMR and cardiac CT offer a more accurate identification and quantification of pericardial thickness given their higher spatial resolution, with abnormal thickening generally defined as exceeding 3–4 mm.^30^ CT is particularly advantageous in identifying calcifications, though it is essential to recognize that pericardial calcification may occur independently of constrictive physiology.^30^ Given the limitations of individual modalities, a multimodal imaging approach is often necessary for a comprehensive evaluation and definitive diagnosis of pericardial pathology.

### 4.2 Pericardial Edema

Pericardial edema results from increased vascular permeability and fluid accumulation within the pericardium secondary to a pathologic insult, signifying an acute or hyperacute pericardial pathology. Although the identification of pericardial edema on imaging supports ongoing inflammation, its absence does not exclude it, as pericardial edema is less commonly appreciated in subacute and chronic inflammatory processes.

CMR is the gold standard for detecting pericardial edema, utilizing T2‐weighted short tau inversion recovery (T2‐STIR) sequences. Crucially, adequate fat suppression techniques must be implemented during image acquisition to prevent misinterpretation. Pericardial edema appears as a hyperintense (bright) signal on T2‐weighted images, with variable distribution. It may partially or circumferentially affect the visceral pericardium, the parietal pericardium, or both layers. The high‐resolution tissue characterization provided by CMR facilitates accurate assessment and grading of the edema, which is vital for the diagnosis, risk stratification, and management of pericarditis.^26^, ^31^ Other imaging techniques are not useful for detecting pericardial edema and are therefore not indicated.^26^, ^31^, ^32^

### 4.3 Pericardial Neovascularization/Inflammation

Under normal physiological conditions, the relatively avascular pericardium does not exhibit enhancement following contrast agent administration since these agents cannot adequately reach and accumulate in the pericardium. However, pericardial injury induces neovascularization, detectable as pericardial enhancement on late gadolinium enhancement (LGE) imaging with CMR as first‐line imaging or with late iodine enhancement (LIE) on dedicated cardiac CT when CMR is not available or contraindicated.

Pericardial LGE on CMR is a highly sensitive (94%–100%), albeit indirect, marker for detecting pericardial inflammation, making it a cornerstone in the diagnosis of pericarditis.^33^ Nonetheless, it is essential to emphasize that this finding reflects current or prior pericardial injury rather than pure active inflammation.^21^ The more severe and intense the enhancement is, the higher the likelihood of acute injury and ongoing inflammation, with pathology studies demonstrating a direct correlation between the extent and intensity of pericardial LGE and the presence of pericardial inflammation, fibroblastic proliferation, and neovascularization. Notably, milder, less intense persistent forms of enhancement may be observed because of past pericardial injury, even in individuals who are currently asymptomatic. Consequently, the qualitative or semi‐quantitative assessment of LGE, which involves categorizing the enhancement as mild, moderate, or severe based on its signal intensity, may provide a more nuanced understanding of the inflammatory process.

Beyond diagnosis, pericardial LGE has been firmly established as a significant prognostic marker, particularly in CP. Notably, moderate to severe pericardial LGE, accompanied by elevated inflammation markers, is strongly associated with reversible CP. Most patients (93%) with reversible constrictive pericarditis exhibit moderate or severe enhancement, in contrast to only 33% in those with persistent constriction.^34^ Additionally, a pericardial LGE thickness of ≥3 mm has been reported to have 86% sensitivity and 80% specificity in predicting the likelihood of reversibility in constrictive pericarditis. Moreover, the resolution or reduction of pericardial LGE following anti‐inflammatory therapy correlates with clinical improvement and the resolution of constrictive physiology. These findings underscore the role of CMR‐derived pericardial LGE, or alternative LIE by cardiac CT as alternative imaging, not only as a key diagnostic tool but also as a prognostic marker, providing critical guidance for treatment decisions and risk stratification in patients with pericarditis.^34^, ^35^

### 4.4 Pericardial Inflammation

Pericardial inflammation is characterized by the infiltration of inflammatory cells into the pericardium. This process is typically accompanied by edema, neovascularization, granulation tissue formation, and fibroblastic proliferation. The inflammatory response can manifest as an acute condition, often dominated by neutrophils and significant edema, or evolve into a more indolent, chronic form. Chronic pericarditis is frequently associated with a different profile of cellular infiltrates, minimal to no edema, and more pronounced tissue remodelling.

18F‐FDG PET‐CT/MR has emerged as a valuable imaging modality for detecting pericardial inflammation and neoplastic processes of the pericardium. The utility of this technique lies in the principle that metabolically active inflammatory and cancer cells exhibit avid uptake of the glucose analog 18F‐FDG. A key advantage of Hybrid 18F‐FDG PET‐CT/MR is its ability to perform whole‐body assessments, which is particularly valuable when there is a suspicion of underlying systemic inflammatory disorders or occult malignancies.^36^ Beyond simply detecting inflammation, Hybrid 18F‐FDG PET‐CT/MR can also quantify the intensity of the inflammatory process by measuring the maximum standardized uptake value (SUVmax). A significantly elevated SUVmax value, in the range of 5.0 to 10.0 or higher, may indicate specific etiologies such as tuberculous or neoplastic pericarditis. Additionally, this modality has demonstrated prognostic utility in predicting the reversibility of transient CP with anti‐inflammatory therapies using an SUVMAx cut‐off of >3.0. Furthermore, serial 18F‐FDG PET‐CT/MR can assess treatment response to anti‐inflammatory therapy by monitoring changes in pericardial metabolic activity over time. This highlights the potential of 18F‐FDG PET‐CT/MR in guiding therapeutic strategies.^37^, ^38^

Despite its benefits, 18F‐FDG PET‐CT/MR has certain limitations in evaluating pericardial disease. One concern pertains to its sensitivity in detecting milder forms of pericardial inflammation when pericardial thickening is absent, which can be attributed to the inherent limitations in spatial resolution of the modality. Furthermore, the diagnostic specificity of 18F‐FDG PET‐CT/MR in differentiating various pericardial pathologies remains imperfect. A significant overlap in SUVmax values, particularly within the range of 1.0–5.0, has been observed between malignant and inflammatory conditions.^38^ Further limitations include the need for strict dietary preparation, the high cost of the procedure, and radiation exposure, making it unsuitable for routine evaluation of all pericardial diseases. Nonetheless, when applied judiciously in complex or equivocal clinical cases, 18F‐FDG PET‐CT/MR provides valuable diagnostic, prognostic, and therapeutic insights, aiding in the comprehensive management of pericardial inflammation.^37^

### 4.5 Pericardial Fibrosis and Calcification

Pericardial fibrosis and calcification represent the advanced stages of pericardial diseases, frequently resulting from a cascade of tissue remodelling processes in response to repetitive or chronic myocardial injury. CMR is the preferred modality for detecting pericardial fibrosis, which may manifest as a thickened pericardium on T1‐weighted imaging, with no or mild persistent enhancement on LGE imaging and the absence of edema on T2‐weighted imaging. Pericardial calcification is characterized on CMR as areas of signal void within the pericardium.^2^ In cases where differentiating mild chronic pericardial inflammation from fibrotic pericardium is challenging, particularly in the presence of mild persistent LGE in a thickened pericardium, 18F‐FDG PET‐CT/MR may help resolve this diagnostic dilemma by demonstrating or excluding underlying active inflammation. On the other hand, cardiac CT is the gold standard for detecting pericardial calcification. Its superior spatial resolution enables the precise localization and quantification of even minimal calcium deposits.^29^, ^30^ This high‐resolution capability is particularly valuable for preoperative planning in constrictive pericarditis cases where accurate delineation of calcified regions is essential for surgical intervention. A comprehensive multimodality imaging approach is often required to provide a thorough assessment of pericardial fibrosis and calcification.

### 4.6 Pericardial Effusion and Tamponade Physiology

PEff, characterized by the accumulation of pericardial fluid exceeding 50 mL, stems from diverse etiologies. AP is often associated with small, rapidly developing effusions. Meanwhile, CTP occurs when pericardial fluid accumulation exceeds pericardial compliance, which results in impaired cardiac filling and chamber compression and, ultimately, hemodynamic collapse.

TTE remains the first‐line imaging modality for evaluating PEff and tamponade physiology, given its widespread availability, rapid diagnostic capability, and ability to assess the hemodynamic consequences of Peff.^29^ This technique effectively detects and quantifies the presence of a PEff as an echo‐free separation between the layers of the pericardium. Furthermore, it can aid in differentiating the nature of the effusion, distinguishing between the anechoic appearance of transudative fluid and the heterogeneous echogenicity characteristic of exudative or complex effusions. TTE plays a critical role in detecting cardiac tamponade, identifying key features such as right ventricular diastolic collapse, right or left atrial systolic collapse, respiratory variations in mitral and tricuspid inflow patterns, and plethoric inferior vena cava^2^ (Table 2).

**TABLE 2:** Imaging findings associated with cardiac tamponade and pericardial constriction on echocardiography along with their respective sensitivities and specificities.

Condition	Imaging findings	Sensitivity	Specificity
Cardiac Tamponade	Pericardial effusion	High (95%)	Low (60%–70%)
	Right atrial systolic collapse	Moderate (75%)	High (90%–95%)
	Right ventricular diastolic collapse	High (90%)	High (85%–90%)
	Dilated inferior vena cava (IVC) with reduced respiratory variation	High (85%–90%)	Moderate (70%–80%)
	Exaggerated respiratory variation in mitral and tricuspid inflow velocities (pulsus paradoxus)	Moderate (70%)	High (90%)
Constrictive Pericarditis	Respiratory septal shift (ventricular interdependence)	High (85%–90%)	High (90%–95%)
	Annulus reversus (reduced mitral annular e’ velocity but preserved or increased lateral annular e’)	Moderate (70%–80%)	Moderate (75%–85%)
	Dilated IVC with reduced inspiratory collapse	High (85%)	Moderate (75–80%)
	Pericardial thickening (occasionally visible)	Moderate (60–70%)	Moderate (70–80%)
	Prominent diastolic flow reversal in hepatic veins	Moderate (75%)	High (90%)

When TTE findings are inconclusive or insufficient for a comprehensive assessment of pericardial effusion, TEE, CMR, or cardiac CT serve as valuable complementary imaging modalities. These advanced techniques offer superior spatial resolution, enabling precise localization and quantification of pericardial effusions, particularly in loculated or regional collections that may be causing focal tamponade. CMR and cardiac CT may offer insight into the nature and causes of the effusion based on fluid T1 and T2 characteristics and density (Hounsfield units), respectively. Cardiac CT can also guide pericardiocentesis in complex scenarios involving small, multiloculated, or difficult‐to‐access effusions. By integrating the strengths of TTE, CT, and CMR, clinicians can achieve greater diagnostic accuracy, improved risk stratification, and optimized management strategies for patients with pericardial effusions with or without tamponade.^4^, ^39^

### 4.7 Constrictive Physiology

CP develops when inflammation, fibrosis, or calcification causes the pericardium to become non‐compliant. This rigid encasement of the heart restricts diastolic filling, ultimately resulting in heart failure. CP is categorized into distinct subtypes. These include transient constrictive pericarditis (TCP), which is characterized by inflammation‐mediated constriction; effusive‐constrictive pericarditis (ECP), where constrictive physiology ensues after pericardiocentesis for CTP; and chronic constrictive pericarditis, characterized by a fibro‐calcific pericardium as a result of bouts of pericarditis among other possible causes.

TTE remains the primary imaging modality for assessing the presence of CP across the spectrum of pericarditis, providing essential insights into its characteristic pathophysiology.^40^ Key echocardiographic findings include ventricular septal bounce, respirophasic septal shift, and preserved or augmented early diastolic mitral annular velocities (e’), which help distinguish constrictive physiology (Table 2). Advanced echocardiographic techniques, such as two‐dimensional (2D) speckle tracking, enhance diagnostic precision by differentiating constrictive pericarditis from restrictive cardiomyopathy by assessing longitudinal and circumferential left ventricular mechanics.^40^, ^41^ In certain instances of non‐diagnostic echocardiography, complementary CMR offers real‐time free‐breathing cine sequence, which allows the detection of respirophasic septal shift, characteristic of CP. Moreover, this modality can also identify the presence of septal bounce, dilated inferior vena cava, pericardial adhesions, tubular deformation of ventricles, and bi‐atrial enlargement.^2^, ^40^ Finally, CMR and/or hybrid 18F‐FDG PET‐CT/MR also serve as a valuable complementary imaging modality to identify TCP via the direct or indirect detection of pericardial inflammation and predict the reversibility with anti‐inflammatory therapies.

## 5 WHEN AND HOW TO USE CARDIAC MULTIMODALITY IMAGING IN PERICARDITIS

Echocardiography, specifically TTE, serves as the first‐line imaging modality for all patients with known or suspected pericarditis. Its primary role is to detect PEff, a supportive finding within the well‐established criteria for diagnosing pericarditis (Table 1). Beyond detection, TTE facilitates initial risk stratification by assessing the size and characteristics of any PEff and identifying potential complications such as CTP and CP. TTE also aids in detecting myocardial involvement, which is revealed through regional wall motion abnormalities and reduced left ventricular systolic function. Moreover, TTE is valuable in guiding pericardiocentesis when indicated and in identifying effusive constrictive pericarditis (ECP) following the procedure.^2^

Despite the established clinical criteria, achieving a definitive diagnosis of pericarditis can be challenging in routine clinical practice, often leading to persistent diagnostic uncertainty. In cases where the diagnosis remains uncertain, complications are suspected, recurrent or incessant pericarditis is present, or therapeutic failure is observed, complementary cMMI is typically warranted. This integrative imaging approach combines echocardiography with CMR, hybrid 18F‐FDG PET imaging, and/or cardiac CT, aiming to address several key objectives: first, to establish a definitive diagnosis of pericarditis by directly or indirectly demonstrating pericardial inflammation; second, to identify complications, including Peff, CTP, CP, or myocarditis; and third, to guide therapeutic management and assess treatment efficacy in patients with incessant, recurrent, or complicated pericarditis.^3^ Table 3 summarizes the key strengths alongside the respective sensitivities and specificities of different imaging modalities in the diagnosis of pericarditis. Furthermore, cMMI enables accurate quantification of the inflammatory burden and comprehensively characterises pericarditis across its stages, from acute and chronic inflammation to fibrosis and remission. A stepwise approach to cMMI is essential, tailored to the individual patient's needs and focused on the specific imaging targets of pericarditis. Each imaging modality's indications, advantages, and limitations (Table 4) must be carefully considered. If initial imaging tests confirm the diagnosis and provide adequate risk stratification and therapeutic guidance, subsequent imaging is usually unnecessary.^2^

**TABLE 3:** Sensitivity and Specificity of Imaging Modalities in Pericarditis Evaluation.

Imaging Modality	Key Strengths	Sensitivity	Specificity
Echocardiography	Readily available; identifies effusion, tamponade, and constriction	~60%–70%	~70%–80%
Cardiac MRI	High soft tissue contrast; detects pericardial edema and LGE (inflammation/fibrosis)	~85%–95%	~80%–90%
Cardiac CT	Excellent spatial resolution; detects calcification and thickening	~70%–80%	~70%–90%
FDG‐PET/CT	Detects metabolic activity; useful for active inflammation	~85%–100%	~80%–90%

**TABLE 4:** The main indications, advantages and disadvantages/ limitations of echocardiography, CT, CMR and hybrid 18F‐FDG CT/MR imaging in the evaluation of pericardial disorders.

Imaging Modality	Indications	Advantages	Limitations
Transthoracic Echocardiography (TTE)	Initial evaluation for pericardial effusion, tamponade, and constriction. Intraprocedural guidance for pericardiocentesis.	Widely available, cost‐effective, portable, and provides real‐time hemodynamic assessment.	Operator and body habitus dependent; limited resolution for posterior pericardial anatomy.
Cardiac Magnetic Resonance (CMR)	Characterization of pericardial inflammation, fibrosis, effusion, and masses. Ideal for tissue characterization and follow‐up in recurrent cases.	High spatial resolution, non‐invasive, no ionizing radiation, and comprehensive evaluation of cardiac and extracardiac structures.	Expensive, wide inter‐observer variability in interpretation, limited availability, contraindicated in certain implants, and requires prolonged imaging times.
Cardiac Computed Tomography (CT)	Evaluation of pericardial calcifications, masses, and effusion. Preoperative planning for pericardiectomy.	High spatial resolution, rapid imaging, and excellent for assessing calcifications and extracardiac anatomy.	Involves ionizing radiation, potential nephrotoxicity from contrast agents, and lower temporal resolution.
Hybrid 18F‐FDG CT/MR imaging	Detection of active inflammation and assessment of treatment response in complex or refractory cases.	Sensitive to inflammation and provides metabolic information beyond structural imaging.	Limited availability, involves ionizing radiation, and lower spatial resolution compared to other modalities.

**FIGURE 3:**
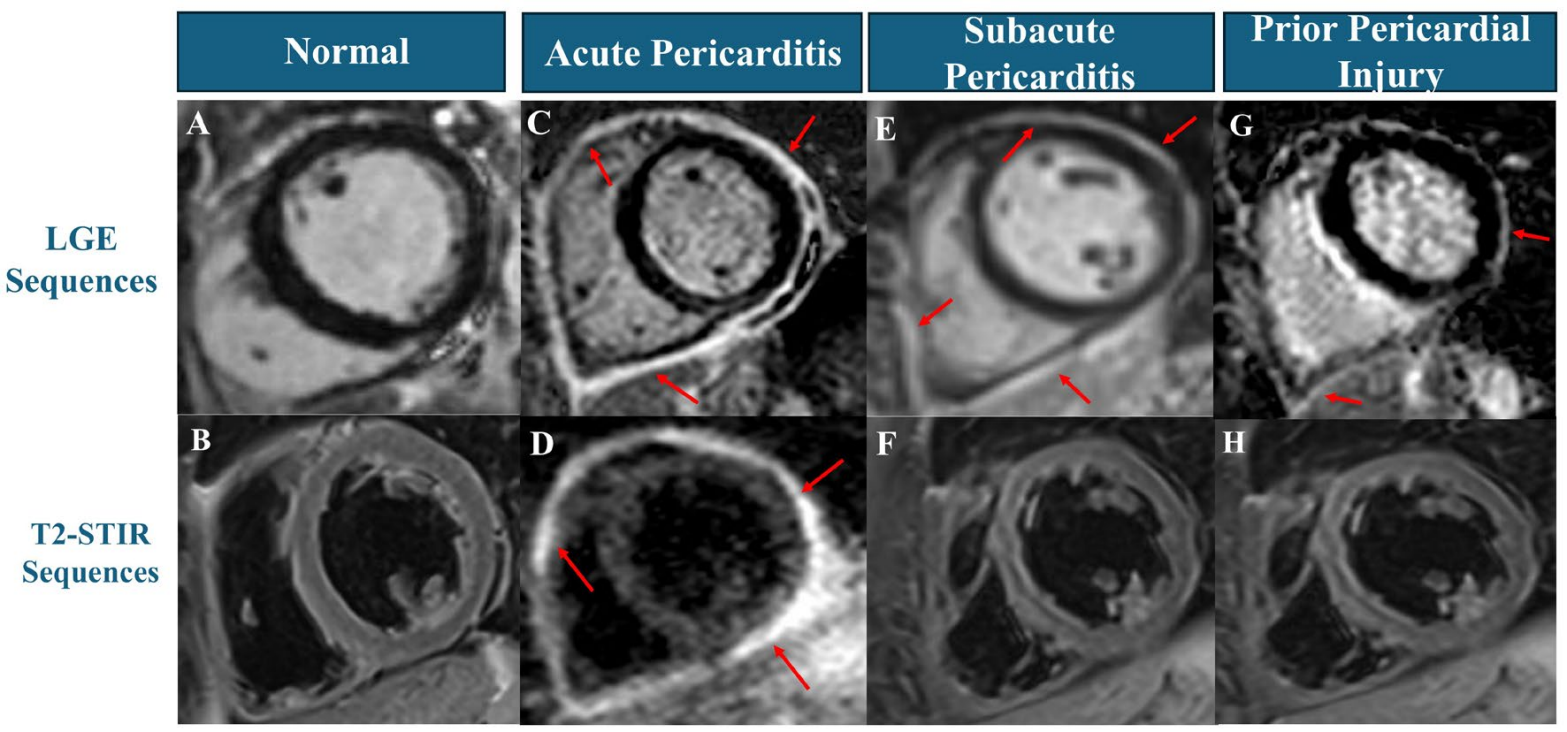
Multimodal Imaging Using CMR Across the Spectrum of Pericardial Diseases. It underscores CMR findings based on late gadolinium enhancement sequences (LGE) and T2‐short tau inversion recovery (T2‐STIR) sequences in a normal patient (panels A and B), a patient with acute pericarditis (panels C and D), a patient with subacute pericarditis (panels E and F) and a patient with prior pericardial injury and no active pericardial inflammation or edema (panels G and H). Red arrows in the LGE sequence row highlight pericardial late gadolinium enhancement, and the red arrows in the T2‐STIR row point to increased pericardial intensity reflective of edema.

When a complementary cMMI approach is required, CMR is the preferred second‐line imaging modality. This modality can accurately diagnose and quantify the severity of pericarditis across its entire spectrum of clinical presentations (Figure 3). Notably, a normal CMR pericardial study is equally informative as it effectively excludes pericarditis. This imaging modality also has the potential to differentiate between subtypes of pericarditis and past pericardial injury with residual fibrosis through integrative evaluation of pericardial thickening, degree of edema, neovascularization, and fibro‐calcific changes, a particularly valuable capability in suspected recurrent pericarditis.^26^ Furthermore, CMR can detect and characterize PEff, ascertain its hemodynamic impact, identify coexisting CP, and evaluate for concomitant myocarditis.^2^, ^42^ Finally, this modality serves as a cornerstone for implementing IGT by guiding the intensity, length, and de‐escalation of therapies based on the burden of LGE as a surrogate for the severity of pericarditis on initial and/or serial imaging. This approach has been shown to decrease the likelihood of recurrence, exposure to steroids, and pericardiocentesis.^43^

When CMR is not feasible or contraindicated, or diagnostic uncertainty remains despite CMR, hybrid 18F‐FDG PET imaging may be a viable alternative to CMR or complementary as a third‐line modality, respectively. Although it requires adequate preparation, this modality has demonstrated the ability to detect active pericardial inflammation through areas of increased radiotracer uptake. Additionally, hybrid 18F F‐FDG PET imaging may provide valuable insights into predicting the response to anti‐inflammatory therapies in cases of TCP.^2^, ^37^ While its sensitivity for detecting milder forms of inflammation in a non‐thickened pericardium is limited, this modality can provide complementary information to CMR in select scenarios of uncertainty about the degree and/or presence of inflammation.^44^, ^45^

Cardiac CT may be considered a third‐line imaging modality when CMR is contraindicated or not feasible, and hybrid 18F‐FDG PET imaging is not available. This modality detects pericardial thickening and LIE, which may indirectly indicate pericardial disease and inflammation. Notably, even non‐dedicated chest CT scans with contrast, performed for other reasons, can reveal pericardial thickening and enhancement in cases of suspected pericarditis and must be carefully reviewed when available. Cardiac CT excels at identifying pericardial calcification as a sequela of prior pericarditis. Moreover, it accurately identifies and characterises PEff, distinguishing PEff from mimics like pleural effusion or pericardial fat. Although cine cardiac CT can assess constrictive physiology, radiation concerns limit its routine clinical use. Finally, cardiac CT comprehensively evaluates extracardiac structures, potentially revealing systemic conditions or secondary causes of pericarditis, such as malignancy.^2^

## 6 IMAGING‐GUIDED THERAPY IN PERICARDITIS

**FIGURE 4:**
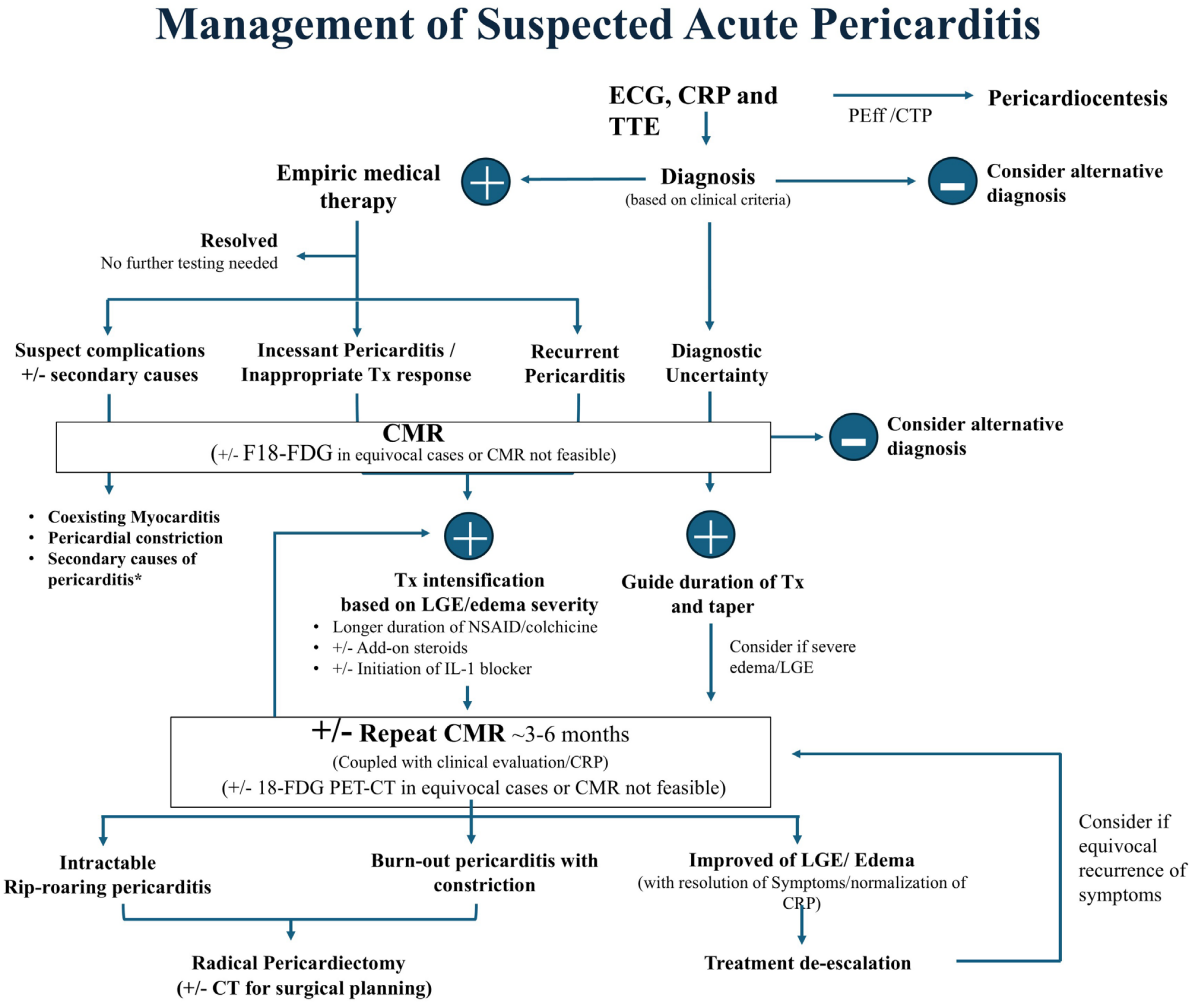
Management Algorithm for suspected Acute Pericarditis and associated sequelae. CMR, cardiac magnetic resonance; CRP, C‐reactive protein; CT, computed tomography; ECG, electrocardiogram; Il‐1, Interleukin‐1; LGE, late gadolinium enhancement; NSAID, non‐steroidal anti‐inflammatory drugs; TTE, transthoracic echocardiography; Tx, treatment.

Over the past decade, the management of pericarditis has undergone a paradigm shift with the introduction of novel anti‐inflammatory therapies, particularly interleukin‐1 (IL‐1) blockers. The emergence of these agents has been instrumental in establishing a framework for targeted, individualized treatment strategies for pericarditis.^46^ Contemporary management strategies for acute and recurrent pericarditis are detailed in Figure 4 and Table 5. The efficacy of IL‐1 blockers in managing recurrent pericarditis is well supported by clinical evidence, especially in cases of colchicine resistance or corticosteroid dependence.^47^, ^48^

**TABLE 5:** Pharmacologic Management of Acute and Recurrent Pericarditis: Drug Classes, Dosage, Considerations, and Adverse Effects.

Medical Therapy	Dosage and Administration	Special Considerations	Common Reported Side Effects
NSAIDs			
Ibuprofen	600–800 mg every 6–8 h	Taper over 2–4 weeks based on symptom resolution. Use with proton pump inhibitors (PPIs) to reduce gastrointestinal risk.	Gastrointestinal upset, ulcers, renal dysfunction, cardiovascular risk
Aspirin	750–1000 mg every 8 h	Preferred in post‐myocardial infarction patients (Dressler syndrome). Taper over 2–4 weeks.	Gastrointestinal upset, tinnitus, bleeding risk
Indomethacin	50 mg every 8 h	Alternative NSAID option. Taper based on clinical response.	Gastrointestinal upset, headache, dizziness, renal dysfunction
Colchicine	0.6–1.2 mg daily (.6 mg twice daily if weight >70 kg)	Administer for at least 3 months in AP and at least 6 months in case of RP.	Diarrhoea, nausea, abdominal pain
Corticosteroids			
Prednisone	0.2–0.5 mg/kg/day	Reserved for cases refractory to NSAIDs and colchicine or contraindications to these therapies. Taper slowly to avoid recurrence.	Weight gain, hyperglycemia, hypertension, osteoporosis, infection risk
Il‐Blockers			
Anakinra	2 mg/kg/day (maximum 100 mg daily) via subcutaneous injection	Consider for refractory recurrent pericarditis or when corticosteroids are contraindicated.	Injection site reactions, neutropenia, increased infection risk
Rilonacept	320 mg loading dose, followed by 160 mg weekly via subcutaneous injection	FDA‐approved for recurrent pericarditis. Demonstrates rapid symptom relief and reduced recurrence risk.	Injection site reactions, upper respiratory infections, hyperlipidemia

*Note*: NSAIDs (Nonsteroidal Anti‐Inflammatory Drugs): First‐line agents for acute pericarditis, reducing inflammation and pain. Require gradual tapering and gastroprotection. Colchicine: Adjunct therapy to NSAIDs, shown to decrease recurrence risk. Requires prolonged administration for optimal efficacy. Corticosteroids: Reserved for cases refractory to NSAIDs and colchicine or in autoimmune pericarditis. Must be tapered slowly to prevent recurrence. IL‐1 Blockers: Targeted biologic agents (Anakinra, Rilonacept) used for refractory or recurrent pericarditis when conventional therapy fails or is contraindicated.

Developments in cMMI have fundamentally changed the approach to pericarditis treatment, leading to the adoption of IGT. This pragmatic approach allows clinicians to tailor treatment initiation, intensification, and duration based on the severity and extent of pericardial inflammation. IGT may also offer an objective measure of treatment efficacy and can help evaluate remission, particularly in challenging or refractory cases. CMR plays a pivotal role in IGT, with key findings serving as prognostic indicators.

Pronounced pericardial edema, visualized on T2‐STIR sequences, and extensive LGE, as an indirect marker for inflammation, are recognised as poor prognostic markers in pericarditis. In a sub‐study of the Rhapsody trial, those with moderate to severe LGE had a shorter time to recurrence of pericarditis (4.2 weeks) than those with mild LGE.^49^ These findings may indicate the need for a more prolonged course of treatment with NSAIDs and colchicine, including a slow and careful tapering of the regimen upon resolution of clinical symptoms, normalisation of inflammatory markers, and resolution or at least marked improvement of CMR findings. The persistence of moderate to severe pericardial LGE on serial CMR imaging despite initial NSAID and colchicine therapy, signifying incessant or “rip‐roaring” pericarditis, may indicate the need to escalate treatment to interleukin‐1 (IL‐1) blockers. On the other hand, the absence of significant LGE on CMR allows for the confident tapering of therapies, particularly glucocorticoids, thus impacting the overall treatment duration and strategy.^34^, ^50^

Furthermore, the burden of LGE, in integration with the level of inflammatory markers and clinical presentation, can be used to predict the reversibility of constrictive physiology in suspected TCP cases. Significant pericardial LGE suggests that active pericardial inflammation is a major contributor to constrictive physiology, and such cases may respond favourably to targeted anti‐inflammatory therapy. In contrast, the absence of enhancement indicates that the constriction is more likely due to advanced fibrosis, a condition unlikely to improve with anti‐inflammatory treatment. In end‐stage pericarditis, characterised by extensive pericardial fibrosis, significant pericardial thickening, minimal vascularity, and minimal to no inflammation, pericardial delayed enhancement is typically absent or minimal.^3^ Finally, in cases of burnt‐out or calcific pericardium with chronic CP, radical pericardiectomy is curative, and pre‐surgical planning with cardiac CT is indispensable.^2^ In cases where the clinical presentation is atypical, such as absence of chest pain, negative inflammatory markers, and a completely normal pericardium on CMR—this combination may help argue against active pericarditis. However, CMR findings should always be interpreted alongside clinical and laboratory data. Recently, data from the RESONANCE study reported that up to 50% of patients with clinically confirmed recurrent pericarditis had unremarkable CMR findings at the time of imaging. Notably, the study lacked serial imaging, core lab interpretation, and did not distinguish between truly normal versus subtly abnormal pericardial findings. It's also unclear whether imaging may have normalised following prior treatment, leading to potential false‐negative results. These findings highlight the limitations of single time‐point imaging and the pitfalls of relying on single modality interpretation in isolation and reinforce the need for prospective studies with standardised CMR protocols, serial assessments, and centralised interpretation to clarify the diagnostic role of CMR in suspected pericarditis.^51^

There are no standardized guidelines regarding the timing of second‐line imaging modalities, particularly CMR. However, when indicated, CMR can be performed at baseline and 3–6‐month intervals to evaluate treatment response and monitor disease progression. Meanwhile, repeat CMR may also be considered with recurrences of pericarditis‐like symptoms in patients with a history of recurrent pericarditis in remission or during therapy tapering to confirm or exclude recurrent pericarditis.^2^ In a study by Bianco et al., the utility of CMR in guiding therapeutic decisions for patients with recurrent pericarditis receiving Anakinra was assessed through longitudinal imaging assessment—particularly of pericardial inflammation and fibrosis markers such as LGE and edema—CMR was shown to aid in evaluating treatment response and informing the duration and tapering of anti–interleukin‐1 therapy. These findings support the integration of CMR as a non‐invasive modality to individualize management strategies and reduce the risk of disease recurrence in this patient population.^52^

## 7 CONCLUSION

Pericarditis encompasses a spectrum of presentations, including acute, recurrent, incessant, and constrictive forms, each with distinct auto‐inflammatory, autoimmune, and non‐inflammatory phenotypes. While traditional diagnostic and management approaches have faced limitations, novel concepts in the pathophysiology of pericarditis, advances in cMMI, and the introduction of novel therapies, including IL‐1 blockade, have revolutionised its care. The advent of IGT, facilitated by cMMI advancements, marks a paradigm shift in pericarditis care. IGT moves beyond reliance on solely clinical criteria, enabling clinicians to tailor treatment – including initiation, intensification, and duration – based on objective evidence of pericardial pathology derived from specific imaging targets. While cMMI plays a central role in the evaluation of pericarditis, each modality has inherent limitations, and findings must be interpreted in the context of clinical presentation and laboratory data. Normal imaging does not definitively exclude disease, particularly in recurrent or treated cases. Variability in timing, image acquisition, and lack of standardised interpretation, especially in real‐world settings, further underscore the importance of a comprehensive, integrated diagnostic approach. Further research is needed to solidify and expand the role of cMMI and IGT in optimising pericarditis management.

## AUTHOR CONTRIBUTIONS

Dr. El Roumi and Dr. Schenone worked hand in hand during the writing process of the paper, including the creation of tables and figures. Dr. El Roumi was also responsible for all citations and references. Dr. Wang assisted in manuscript revision and figures/tables optimization. Drs. Cremer and Klein reviewed the manuscript as a whole and provided feedback and comments for the first and second authors to tackle and adjust.

## FUNDING INFORMATION

Dr Klein has received research grants from Kiniksa Pharmaceuticals and Cardiol therapeutics and Ventyx, and is on advisory boards for Kiniksa Pharmaceuticals, Cardiol Therapeutics, Ventyx and Pfizer. Dr Cremer has received research grants from Kiniksa Pharmaceutics and Novartis and is on advisory boards for Kiniksa Pharmaceutics and Swedish Orphan Biovitrum. Dr Schenone is on the speaking bureau for Bristol Myers Squibb. The rest of the authors report no relationships relevant to the contents of this paper to disclose.

## CONFLICT OF INTEREST STATEMENT

The authors have no relevant conflicts of interest to disclose for this manuscript.

## Data Availability

This is a narrative review article. No new datasets were generated or analysed during the current study. All data and references supporting the findings of this review are available in the published literature, which has been appropriately cited throughout the manuscript.
